# Prostate cancer cells of increasing metastatic potential exhibit diverse contractile forces, cell stiffness, and motility in a microenvironment stiffness-dependent manner

**DOI:** 10.3389/fcell.2022.932510

**Published:** 2022-09-19

**Authors:** Clayton W. Molter, Eliana F. Muszynski, Yuanyuan Tao, Tanisha Trivedi, Anna Clouvel, Allen J. Ehrlicher

**Affiliations:** ^1^ Department of Bioengineering, McGill University, Montreal, QC, Canada; ^2^ Department of Neuroscience, McGill University, Montreal, QC, Canada; ^3^ Department of Electrical and Computer Engineering, McGill University, Montreal, QC, Canada; ^4^ Department of Anatomy and Cell Biology, McGill University, Montreal, QC, Canada; ^5^ Rosalind and Morris Goodman Cancer Research Institute, McGill University, Montreal, QC, Canada; ^6^ Department of Biomedical Engineering, McGill University, Montreal, QC, Canada; ^7^ Department of Mechanical Engineering, McGill University, Montreal, QC, Canada

**Keywords:** prostate cancer, biophysics, microenvironment stiffness, contractility, cell mechanics, cell stiffness, motility, traction force microscopy

## Abstract

During metastasis, all cancer types must migrate through crowded multicellular environments. Simultaneously, cancers appear to change their biophysical properties. Indeed, cell softening and increased contractility are emerging as seemingly ubiquitous biomarkers of metastatic progression which may facilitate metastasis. Cell stiffness and contractility are also influenced by the microenvironment. Stiffer matrices resembling the tumor microenvironment cause metastatic cells to contract more strongly, further promoting contractile tumorigenic phenotypes. Prostate cancer (PCa), however, appears to deviate from these common cancer biophysics trends; aggressive metastatic PCa cells appear stiffer, rather than softer, to their lowly metastatic PCa counterparts. Although metastatic PCa cells have been reported to be more contractile than healthy cells, how cell contractility changes with increasing PCa metastatic potential has remained unknown. Here, we characterize the biophysical changes of PCa cells of various metastatic potential as a function of microenvironment stiffness. Using a panel of progressively increasing metastatic potential cell lines (22RV1, LNCaP, DU145, and PC3), we quantified their contractility using traction force microscopy (TFM), and measured their cortical stiffness using optical magnetic twisting cytometry (OMTC) and their motility using time-lapse microscopy. We found that PCa contractility, cell stiffness, and motility do not universally scale with metastatic potential. Rather, PCa cells of various metastatic efficiencies exhibit unique biophysical responses that are differentially influenced by substrate stiffness. Despite this biophysical diversity, this work concludes that mechanical microenvironment is a key determinant in the biophysical response of PCa with variable metastatic potentials. The mechanics-oriented focus and methodology of the study is unique and complementary to conventional biochemical and genetic strategies typically used to understand this disease, and thus may usher in new perspectives and approaches.

## 1 Introduction

Metastasis is characterized by cancer cell migration from a primary tumor to a new secondary site, and this phenomenon accounts for 90% of cancer-related deaths (Seyfried and Huysentruyt, 2013). Although cancer progression and metastasis are associated with a diverse array of mutations, it seems that in many different cancers, cells change their biophysical characteristics (Northcott et al., 2018). Specifically, the softening of cancer cells’ actin cortex (Cross et al., 2007; Luo et al., 2016) and increased contractility (Kraning-Rush et al., 2012) are emerging conserved biomarkers of metastatic progression, which may facilitate migration through crowded multicellular environments. These mechanics are also influenced by microenvironment stiffness; for example, matrix stiffening resembling changes in tumor microenvironment biases metastatic cells to contract more strongly (Kraning-Rush et al., 2012), promoting tumor phenotypes (Ishihara et al., 2017). Likewise, invasive cellular transformations associated with metastatic and resistant prostate cancer progression, such as the epithelial-to-mesenchymal transition (EMT), have been characterized to undergo robust changes in their contractility (Yoshie et al., 2019), cortical stiffness (Schneider et al., 2013), bulk monolayer mechanics (Sutton et al., 2021), and motility (Leggett et al., 2021).

These frequently observed biophysical trends in cancer cells, however, are not necessarily universal. Prostate cancer (PCa), a very common latent health hazard for men accounting for 10% of male cancer-related deaths (LeBlanc et al., 2019), appears to deviate from the cell softening trends described in other cancer models as PCa cells stiffen within increasing metastatic potential (Faria et al., 2008; Bastatas et al., 2012; Lekka et al., 2012; Khan et al., 2018; Liu et al., 2019). Understanding the mechanisms and functional consequence associated with these deviations remains elusive. However, the mechanical transitions of PCa during metastatic progression are a growing subject of clinical importance. Aggressive and invasive metastatic prostate cancer coincides with the development of resistance to standard therapeutic strategies (Wang et al., 2018; Formaggio et al., 2021), namely, androgen deprivation therapy (ADT) (Zhu and Kyprianou, 2010; Sun et al., 2012; Huo et al., 2015; Miao et al., 2017), making mechanical perspectives valuable in otherwise intractable conditions.

Prior works have generally examined individual aspects of PCa biophysics, such as stiffness (Bastatas et al., 2012; Lekka et al., 2012) or contractility (Kraning-Rush et al., 2012), but have not characterized these together, nor as a function of metastastic potential or microenvironment mechanics. PCa research generally uses several established model metastatic cell lines. Most prominent among these are PC3, DU145, and LNCaP which are known to be being highly, moderately, and lowly metastatic, respectively (Wu et al., 2013)_._ These three model cell lines are also derived from different metastatic sites, each with unique mechanical environments: stiff bone tissue (PC3) (Kaighn et al., 1979), relatively soft brain (DU145) (Stone et al., 1978), and soft lymph node metastases (LNCaP) (Horoszewicz et al., 1980). These may confer unique intrinsic mechanosensitivities and mechanical adaptations. Indeed, the mechanical microenvironment is important in the biology and biophysics of prostate cancer cells. For example, only PC3 cells have been found to exhibit substrate stiffness-dependent phenotypic switching (Aw Yong et al., 2017), including morphology (Prauzner-Bechcicki et al., 2015), cytoplasmic compliance (Baker et al., 2009), proliferation (Liu et al., 2020b), and migration (Yeoman et al., 2021). Similar effects have also been observed independently for LNCaP (Sieh et al., 2012) and DU145 (Prauzner-Bechcicki et al., 2015; Aw Yong et al., 2017). Furthermore, although highly metastatic PC3 cells have been shown to exert higher forces than primary epithelial cells in a stiffness-dependent manner (Kraning-Rush et al., 2012), no study to date has quantified how cell contractility changes with increasing metastatic potential. This is potentially a critical property to characterize, as a recent study found that highly metastatic (PC3) and lowly metastatic (LNCaP) cell lines possess higher metastatic potentials *in vivo* after culture on substrates resembling the microenvironmental stiffness of the site from which they were originally harvested (Liu et al., 2020b). It is to be noted that this is independent of the absolute substrate stiffness magnitude, counter to conventional understandings on the role of microenvironment stiffness in promoting cancer phenotypes.

These confounding results illustrate that microenvironment mechanics are a significant yet nuanced factor in the progression of prostate cancer. A quantitative biophysical characterization may provide valuable insight into how these factors inform directed cell migration toward metastasis. In this study, we describe the interplay among cell mechanics, microenvironment mechanics, and metastatic prostate cancer progression by quantifying cell contractility, cell stiffness, and cell motility. We found that the biophysics of different metastatic potential cells are diverse and do not necessarily scale as a function of metastatic potential, but rather they exhibit unique biophysical responses across a range of microenvironment stiffening similar to that *in vivo*. The results reported here emphasize the need to consider the role of microenvironment as it affects the fundamental mechanical behavior and observed trends across various model cell types when looking to establish mechanical biomarkers for cancer progression.

## 2 Materials and methods

### 2.1 Cell culture

In this study, we conducted a complete quantitative characterization of the putative prostate cancer progression cell lines of PC3 (highly aggressive), DU145 (moderately metastatic), LNCaP (lowly metastatic), and 22RV1 (tumorigenic but not metastatic). The high, moderate, and low metastatic potential classifications have been widely reported in the PCa literature (Wu et al., 2013). These classical definitions are primarily based on each cell line’s ability to metastasize *in vivo*, although the relative invasiveness is often reflected in *in vitro* migration and invasion assays (Supplementary Table S1). To facilitate nuclear tracking, PC3, DU145, and 22RV1 stably expressing mTurquoise-tagged histone H2B protein were generated using lentiviral transduction as a generous gift from the laboratory of Dr. Arnold Hayer. Cells were cultured in RPMI-1640 culture media without phenol red (Wisent Inc.), supplemented with 10% fetal bovine serum (Wisent Inc.) and 1% penicillin–streptomycin (P/S, Wisent Inc.); 0.5 ug/mL puromycin was used in culture media to select for mTurquoise-labelled nuclei. Cells were passaged and used for experiments when cultures reached 70–90% confluency. Prior to cell plating, cells were either dyed with CellTracker Orange for time course TFM/motility assays or CellTracker Green (Invitrogen) for OMTC experiments.

### 2.2 Synthesis of compliant silicone substrates

Compliant polydimethylsiloxane (PDMS) substrates of known stiffness were prepared as previously described (Yoshie et al., 2019; Ghagre et al., 2021). In brief, parts A and B NuSil 8100 (NuSil Silicone Technologies, United States) were at 1:1 w/w ratios. To tune the final substrate stiffness, different concentrations of Sylgard 184 PDMS cross-linking agent (dimethyl, methyl hydrogen siloxane, containing methyl-terminated silicon hydride units) were combined with the PDMS mixture. The mechanical properties of PDMS at the selected cross-linker concentrations have been extensively characterized in the past work (Yoshie et al., 2019; Ghagre et al., 2021). For our experiments, 50 μl of uncured PDMS was applied to 22 × 22 mm (no. 1) glass coverslips and cured at 100°C for 1 h to produce substrates with 100 μm thickness. Depending on the cell membrane label, DII (red, for OMTC)- or DID (far-red, for time course TFM)-conjugated fluorescent fiduciary beads were synthesized as previously described (Klein et al., 2003), coated onto the substrates using a spin coater (WS-650 Spin Processor, Laurell Technologies), and incubated for 1 h for 100°C. Substrates were then fastened to the bottom of a 6-well or 35 mm plates for protein coating and imaging.

The selected substrate stiffnesses include the following: (i) 1 kPa (0.1% w/w Sylgard 184) which resembles the soft lymph node and brain tissue from which LNCaP and DU145 were, respectively, sourced (Liu et al., 2020b); (ii) 3 kPa (0.15% w/w Sylgard 184) which resembles normal prostate tissue (Hoyt et al., 2008; Rouvière et al., 2017); (iii) 12 kPa (0.36% w/w Sylgard 184) which resembles the stiffened prostate tumor microenvironment (Hoyt et al., 2008; Rouvière et al., 2017), and (iv) 50 kPa (0.85% w/w Sylgard 184) which resembles non-mineralized bone environment from which PC3 were originally sourced (Engler et al., 2006); 25 kPa substrates (0.5% w/w Sylgard 184) were also fabricated as an intermediate stiffness between 12 kPa and 50 kPa for the OMTC analysis experiments. This stiffness range bounds stiffnesses used in the previous literature investigating the stiffness-dependent EMT phenotypic switching of PC3 and LNCaP (1 kPa–50 kPa) (Liu et al., 2020b).

### 2.3 Measuring cell contractility using traction force microscopy

Cell-generated surface displacements, traction stress (RMST), and strain energy were quantified with traction force microscopy (TFM) as previously described (Yoshie et al., 2018; Yoshie et al., 2019), using an open-source Python TFM package (pyTFM) modified to the case of cell monolayers and force imbalances within the field of view (Butler et al., 2002; Trepat et al., 2009; Bauer et al., 2021). In brief, surfaces were functionalized using Sulfo-SANPAH (Sigma-Aldrich, MO, United States) as previously described and coated with 5 μg/ml fibronectin to facilitate cell attachment at 4°C overnight (Yoshie et al., 2018; Yoshie et al., 2019). Fibronectin was selected as an adhesive ligand, as it has previously been demonstrated to promote similar adhesion dynamics across lowly metastatic (LNCaP) and highly metastatic (PC3) cell lines compared to other ligands like collagen, which differentially favor PC3 adhesion (Hall et al., 2006; Docheva et al., 2010). Prior to cell plating, cells were labelled with CellTracker Orange CMTMR Dye (Invitrogen; Thermo Fisher Scientific, Inc.). Cells were then allowed to settle on the surface and form monolayers over 48 hours prior to imaging. In this study, we identified monolayers as the interior of continuous multicellular regions providing full coverage of the optical field with high cell density and cell–cell contact. For imaging, cells were mounted on a confocal microscope (Leica TCS SP8 with a 10×0.4 NA objective). During imaging, cells were maintained at 37°C (stage heater, Cell MicroControls, VA) and 5% CO_2_ (perfusing 100% humidity prebottled 5% CO_2_ in synthetic air). Cells, nuclei, and fiduciary TFM bead displacements were simultaneously imaged using fluorescent and transmission microscopy over a several hour time course at time intervals of ∼10–20 min. Experimental images were corrected for lateral drift in ImageJ before being analyzed in the pyTFM workflow for the computation of displacements, traction stress, and monolayer contractile work.

### 2.4 Measuring cell stiffness using optical magnetic twisting cytometry

We quantified the apparent moduli of cells’ actin cortex, henceforth referred to as cell stiffness or cortical stiffness, using optical magnetic twisting cytometry (OMTC). Sample preparation was the same as described for TFM, except a different pairing of fiduciary fluorescent beads and cell dye were used (CellTracker Green with DII beads); 5 μm ferromagnetic beads (Jeff Fredberg Lab, Harvard School for Public Health, United States) were prepared by incubating 21 ug/mL of Arg-Gly-Asp (RGD) peptide (Sigma-Aldrich, MO, United States) in carbonate buffer (15 mM Na_2_CO_3_, 35 mM NaHCO_3_, pH 9.4) overnight at 4°C before being resuspended in PBS. Cells were incubated for 20 min with RGD-coated beads at 37°C and 5% CO_2_. Unattached particles were removed by rinsing with PBS, and then replaced with cell media (RPMI 1640 with 10% FBS and 1% P/S). For imaging, samples were mounted into a set of twisting coils connected to the OMTC device (EOL Eberhard, Switzerland). We applied an oscillating specific torque (defined as the mechanical torque per unit bead volume with dimensions of stress, Pa) with an amplitude of 110 Pa and frequency of 1 Hz. Only singular beads oscillating uniformly were considered. By observing the displacement of the oscillating bead under a known torque and assuming a bead embedding ratio of 10% (Fabry et al., 2003), we calculated the apparent moduli (cell stiffness) as previously described (Fabry et al., 2003; Lenormand et al., 2004; Li et al., 2017). In brief, bead trajectories were determined using a particle tracking plugin, TrackMate, in ImageJ, after which a custom MATLAB program was used to fit a sinusoidal curve to determine the bead displacement amplitude. The amplitude of the fitted curve was then used to determine the apparent modulus. This workflow is illustrated in Figures 2A–C.

### 2.5 Cell motility analysis

Cell motility was determined by tracking fluorescently tagged nuclei (H2B-mTurquoise) concomitantly with the acquisition of time-course traction force microscopy images. First, the raw images were preprocessed by rescaling the intensity and as non-local means denoising. Then, the nuclei were segmented by minimizing the global cross-entropy between the pixel intensity and the average intensity after the segmentation, for the foreground and background, respectively. The nuclei were tracked based on the amount of spatial overlap between the segmented nuclei in consecutive frames (all implemented through CellProfiler 3.1.9) (McQuin et al., 2018). A time lag of half of the duration of each tracked nucleus trajectory was used to compute the mean squared displacement (MSD). Trajectories longer than 15 microns were used to compute the tortuosity. Tortuosity indicates the linearity of the path, and is defined as the ratio of the total trajectory length and the net straight-line distance between the beginning and end of the trajectory. We report the inverse tortuosity, which indicates the persistence of a given cell trajectory, where one indicates a completely straight trajectory and approaching zero is an infinitely curved trajectory.

## 3 Results

### 3.1 Prostate cancer monolayer contractile work varies non-monotonically with substrate stiffness

**FIGURE 1:**
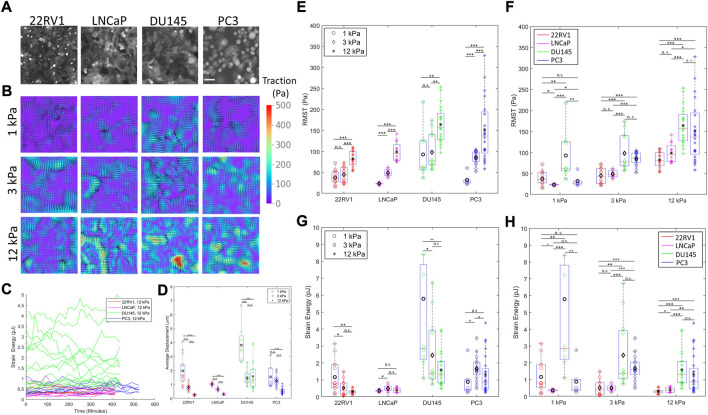
Prostate cancer cells with varying metastatic potential display unique substrate stiffness-dependent monolayer contractility. **(A)** Representative prostate cancer (PCa) monolayers of increasing metastatic potential (22RV1, LNCaP, DU145, and PC3) on 3 kPa substrates labelled with CellTracker Orange CMTR cell dye. **(B)** Representative traction maps generated by monolayers of increasing metastatic potential on substrates of increasing stiffness. **(C)** Representative time traces of measured contractile metrics (here strain energy) for individual monolayers. **(D)** Time-averaged surface displacement exerted by the cell monolayer to its underlying substrate, as measured using a traction force microscopy cross-correlation algorithm. Average displacement measurements converged to similar values approaching the noise level on 50 kPa substrates, suggesting that the 50 kPa substrate is too stiff to resolve cell deformations, restricting our analysis to contractility on 1–12 kPa. **(E–F)** Root mean-squared traction (RMST) stress of PCa cell lines cultured on substrates of various substrate stiffnesses, grouped according to cell type **(E)** and stiffness **(F)**. **(G–H)** Strain energy of PCa cell lines cultured on substrates of various substrate stiffnesses, grouped according to cell type **(G)** and stiffness **(H)**. Scale bar in **(A)** is 50 μm. Black markers on **(D–H)** indicate the mean values. Statistics were calculated using a pairwise Mann–Whitney U test where *p < 0.1; **p < 0.01; ***p < 0.001. Pairwise statistical comparisons across all groups for average displacement, RMST, and strain energy may be found in Supplementary Tables S2, S3, and S4, respectively.

The model cell lines exhibit distinct morphologies upon forming monolayers (Figure 1A). 22RV1 and LNCaP tend to form cohesive monolayers, whereby it becomes difficult to discern one cell from the next. DU145 also forms cohesive monolayers, although its independent cobble-stone-like morphology is easier to be distinguished. In terms of morphology, PC3 cells appear the most morphologically heterogeneous, with a variety of spread areas and different geometries. As such, uniquely during migration, PC3 monolayers frequently develop temporary voids through which other cells can migrate, whereas others move collectively as clusters or by sliding along their neighbors (Supplementary Videos S1–S4).

Cell biophysics and contractility vary as a function of both (i) substrate stiffness and (ii) metastatic potential. Combining both (i) and (ii), we can examine how progressively metastatic cells differentially adjust their contractile behavior in response to different mechanical microenvironments. To quantify cell contractility, we use two metrics describing their contractility—the root mean squared (average) traction stress (RMST, Pascals) and the strain energy (contractile work, in Joules). Traction stress denotes the stress exerted by the monolayer to the substrate, and the strain energy describes the total energy the monolayer commits to deforming the underlying substrate in the form of contractile work (Butler et al., 2002).

With respect to (i), all cell lines displayed less deformation of stiffer substrates (Figure 1D), converging to similar deformation values approaching the noise level on 50 kPa substrates. This suggests that the 50 kPa substrate is too stiff for us to resolve cell deformations. Therefore, our discussion here will be limited to contractility trends observed from 1 to 12 kPa. All cells also increase their traction stress with the increasing substrate stiffnesses (Figures 1B,E), which is a generally expected cell behavior (Califano and Reinhart-King, 2010). Nevertheless, differences in the strain energy as a function of substrate stiffness emerge between the cell lines; 22RV1 (tumorigenic) decrease their strain energy with increasing substrate stiffness, and this inverse trend is mirrored in the reduction of DU145s peak strain energies, although a large range of strain energies with similar median values were maintained for DU145 in the 3–12 kPa stiffness regime (Figures 1G,H). Conversely, PC3 monolayers exhibited a peak in their median strain energy at 3 kPa, increasing from 1 kPa and then decreasing once more. Similarly, LNCaP exhibited a peak in their strain energy on 3 kPa (Figure 1G). These uncorrelated trends between traction stress and strain energy only arise when comparing contractility across different substrates, and not within identical stiffnesses which otherwise universally show positive correlations for both specific monolayers and individual single cells (Supplementary Figure S1A–C). Reduction in contractile work despite increased traction forces with substrate stiffening has been previously reported both in monolayer (Pasqualini et al., 2018) and single cell studies (Müller and Pompe, 2015). These data demonstrate the value of quantifying contractile work in resolving the substrate-stiffness response of cells.

**FIGURE 2:**
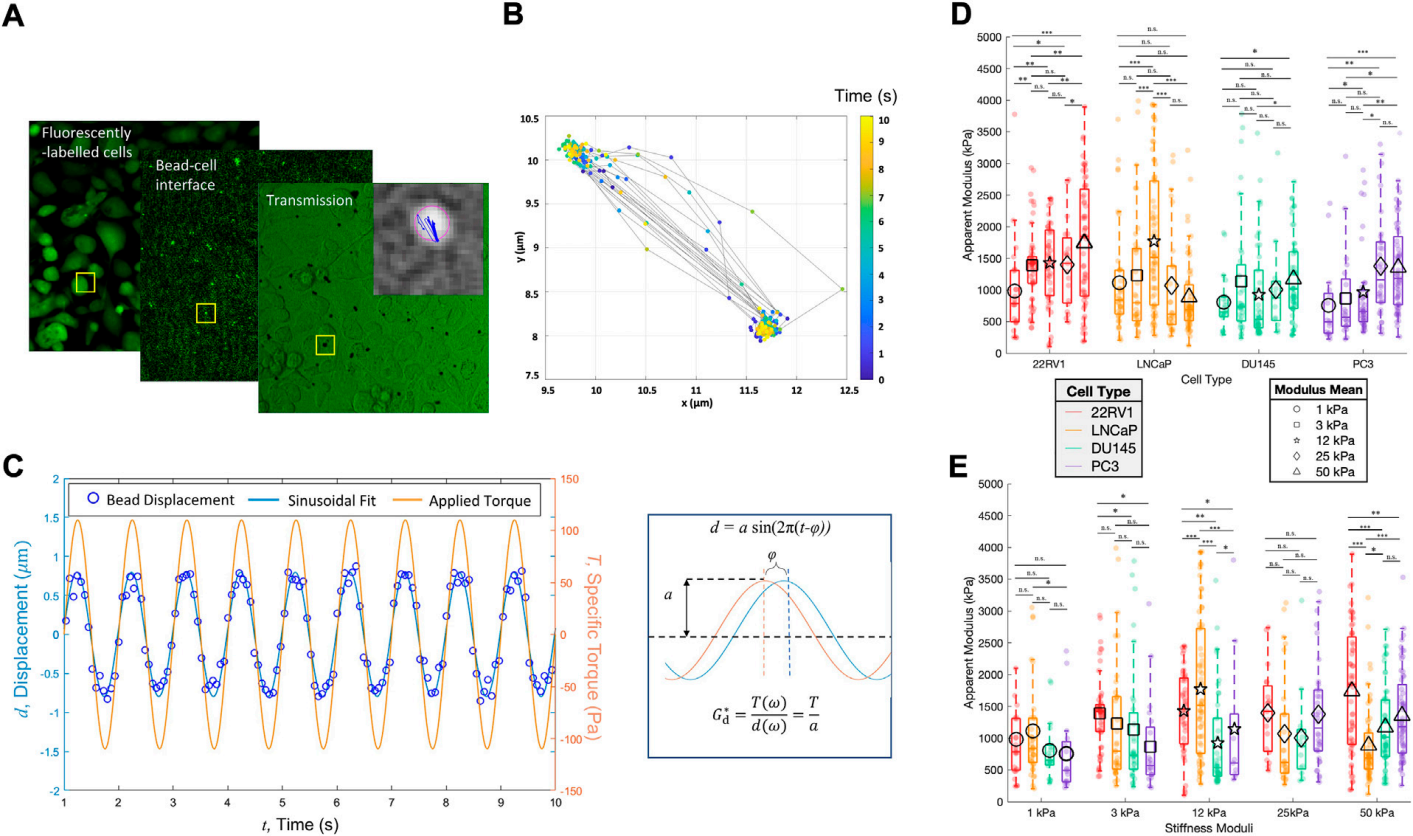
Prostate cancer cell monolayers of varying metastatic potential display diverse cell stiffness. **(A)** Parallelized acquisition of membrane-bound 4.5 um RGD-coated magnetic beads displaced by an oscillating applied torque. The sample shown is PC3 plated on 3 kPa. **(B)** Trajectories of individual beads tracked and synchronized with the applied torque, after which **(C)** bead displacements were fitted to a sinusoid curve to determine the apparent cell modulus, *G*_d_^*^. **(D)** Apparent modulus measured for cell lines of increasing metastatic potential on substrates of increasing stiffness, grouped according to cell type. **(E)** Same data shown in A but clustered according to the substrate type. Statistics were calculated using a pairwise Mann–Whitney U test where *p < 0.1; **p < 0.01; ***p < 0.001. Pairwise statistical comparisons across all groups for apparent cell moduli may be found in Supplementary Table S5.

Comparing different cell types on 1 kPa substrates, the magnitude of traction stress and strain energy appeared unrelated to their relative metastatic potential (Figures 1F,H). At stiffnesses resembling prostate epithelial tissues (3 kPa) and tumor tissue (12 kPa), LNCaP exhibits the lowest substrate displacements, traction stress, and strain energy among metastatic cell lines, with median strain energy comparable to that of the non-metastatic 22RV1 (Figures 2G,H). It is to be noted that the aggressive cell lines of PC3 and DU145 demonstrate a pronounced increase in strain energy and traction stress compared to LNCaP and 22RV1 on 3 kPa and 12 kPa substrates, respectively. This result is consistent with the previously reported positive correlation among contractility, metastatic potential, and substrate stiffening (Kraning-Rush et al., 2012). However, we report that DU145 displays the highest peak strain energies across all cell types (Figures G–H) for 1 and 3 kPa substrates. Furthermore, across all stiffnesses, the median strain energy of DU145 is higher than or similar to that of PC3—the most aggressive cell line. These findings were surprising, given that conventional biophysics knowledge predicts a more metastatic cell line and is expected to be more contractile (Kraning-Rush et al., 2012).

### 3.2 Mechanical microenvironment of source tissue does not predict stiffness-dependent strain energy trends of metastatic prostate cancer

These PC metastatic cell lines were sourced from distinct metastatic sites with different stiffnesses. As a result, the cell lines may have mechanical adaptations toward microenvironment stiffnesses resembling their source metastatic site, which may influence their metastatic capacity (Liu et al., 2020b). LNCaP and DU145 were sourced from metastatic sites with relatively low tissue stiffness (∼1 kPa), yet are dissimilar in contractility (Figures 1F,H), and have different trends in strain energy with an increasing stiffness. In addition, LNCaP and PC3, the latter having been sourced from a relatively stiff bone metastatic site, have strain energies more similar to each other than DU145 at ultrasoft (1 kPa) stiffnesses. The disparity between DU145 and LNCaP, and the intermittent similarity between DU145 and PC3 across all stiffnesses suggest that the potential factor of source tissue stiffness cannot be reconciled using traction force microscopy alone.

### 3.3 Metastatic prostate cancer cells of varying metastatic potential exhibit unique thresholds to which they differentially change their cortical stiffness

The apparent moduli (cell stiffness) of the different PC cell lines vary with the substrate stiffness. 22RV1 illustrated two substrate stiffness thresholds over which noticeable increases in apparent cell stiffness (apparent complex shear modulus G*) were observed (Figure 2). LNCaP demonstrated a single peak in apparent modulus at 12 kPa, but otherwise demonstrated comparable median cell moduli (Figure 2D). This unique peak at tumor-like substrate stiffness indicates a tight range of substrate stiffness over which LNCaP significantly modulates its biophysics. Comparatively, the median stiffness of DU145 is relatively insensitive to changes in substrate stiffness, although there is a slight increase in the apparent modulus when comparing the 1 kPa and 50 kPa groups. Last, PC3 shows a pronounced increase in apparent cell modulus across a threshold between 12 and 25 kPa, before and after which the median stiffnesses were comparable (Figure 2D). Across all cell types and stiffnesses, we identify instances of both high and low apparent moduli (Figures 2D,E), which is likely a consequence of the heterogenous cell morphologies and independent mechanical states within the monolayer. These measurements were conducted in combination with TFM to verify the cells’ contractility; as actomyosin-driven contractile prestress may affect the cells’ cortical stiffness (Schierbaum et al., 2019; Chowdhury et al., 2021). Monolayers sampled for cell stiffness measurements were found to produce strain energies and traction stresses similar to the ranges presented in Figure 1 for the time course experiments, and displayed qualitatively similar stiffness-dependent trends (Supplementary Figure S2).

We observe diverse trends of apparent cell modulus as a function of metastatic potential, whereas at 3 kPa, there is a slight decrease in the mean and/or median cell moduli as a function of metastatic potential; this trend is reversed, leading to cell stiffening for the metastatic cell lines plated on 50 kPa (Figure 2E). Conventional biophysics knowledge predicts cell stiffness to decrease as a function of increased metastatic potential (Luo et al., 2016; Gensbittel et al., 2021). Counter to this trend, the lowly metastatic LNCaP cell line has previously been found to be softer than its more metastatic counterparts (Bastatas et al., 2012; Liu et al., 2019). In our study, LNCaP is only measured to have the lowest median apparent modulus on 50 kPa substrates (Figures 2D,E). However, when adhered to intermediate stiffness substrates, particularly that matching the stiffness of the tumor microenvironment, we observed a pronounced increase in the apparent modulus of LNCaP. Here, LNCaP’s median cell stiffness is found to be more than that of both DU145 and PC3, thus restoring the expected phenotype and suggesting that substrate properties are a key determinant in cell mechanics. We suggest past characterizations of LNCaP as the softest metastatic cell line may have been influenced by measuring their mechanics on substrates with extremely high, supraphysiological stiffness such as glass.

### 3.4 Speed and directional persistence of diverse metastatic potential prostate cancer cells are differentially affected by substrate stiffness

**FIGURE 3:**
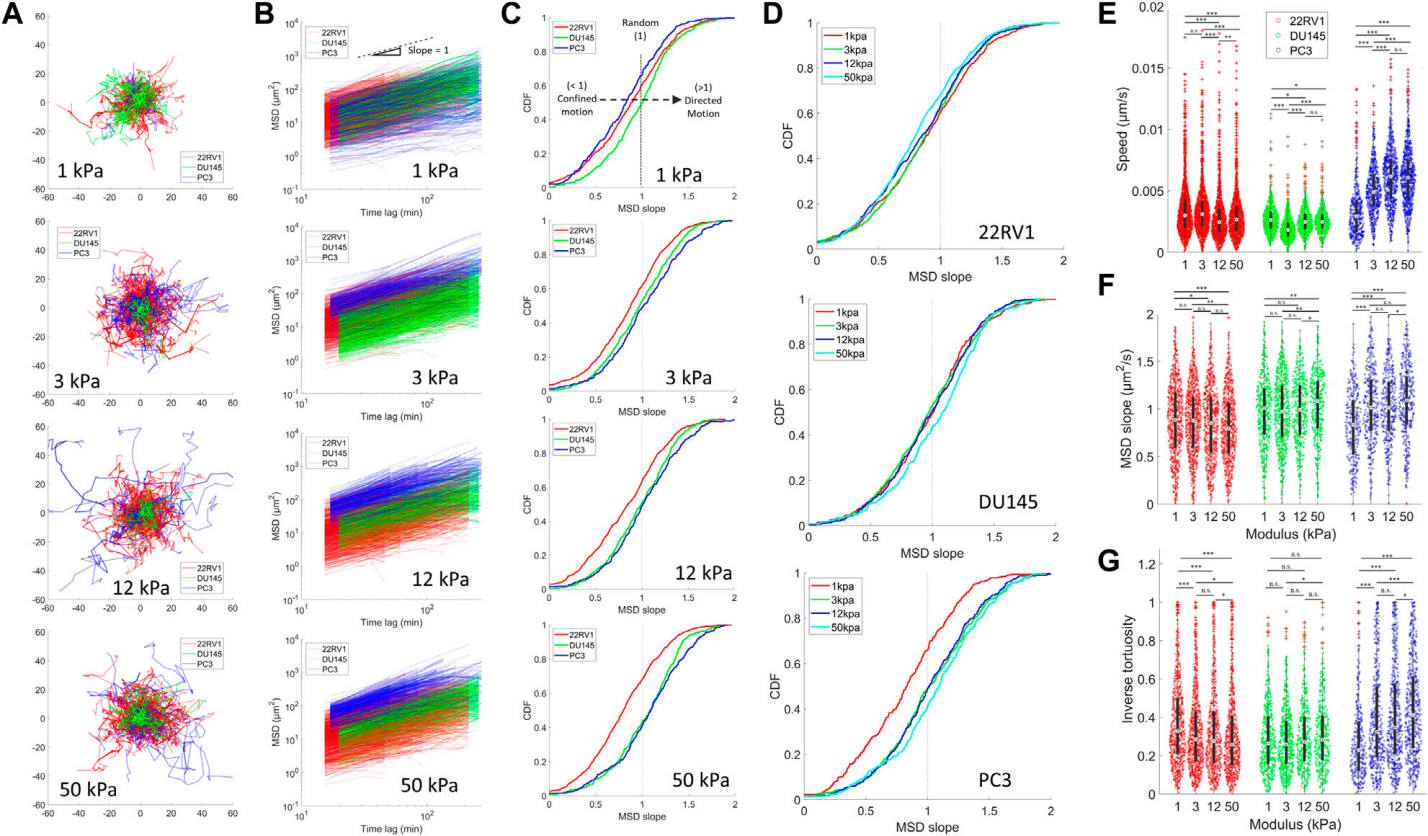
Motility is differentially affected by substrate stiffness for PCa monolayers of varying metastatic potential. Cell motility within a monolayer was measured for PCa cell lines plated on different substrate stiffnesses by tracking fluorescently labelled nuclei. **(A)** Trajectories of PCa cell nuclei on different stiffnesses. **(B)** Mean squared displacements (MSD) as a function of time lag. The black dotted line indicates an MSD slope of one. **(C)** Cumulative probability density functions (CDF) of MSD of slopes calculated from the MSD-time-lag curves for all cell lines on each stiffness (Top–down: 1, 3, 12, and 50 kPa). MSD slopes indicate the nature of cell motion, where slope >1 indicates directed motion, ∼1 indicates random motion, <1 indicates caged or constrained motion, and 0 indicates no motion. Rightward shifting of the CDF curves past a value of 1 indicates a larger proportion of cells undergoing directed motion, as indicated by the black dashed arrow in the top panel. **(D)** CDF with the same data as shown in **(C)** but grouped according to cell type to display stiffness-dependent changes. **(E)** Average cell speed, **(F)** MSD slopes, and **(G)** inverse tortuosity of cells plated on various substrate stiffness as determined by tracking cell nuclei. Inverse tortuosity indicates the persistence of a given cell trajectory, where 1 indicates a completely straight trajectory, and approaching zero is an infinitely curved trajectory. We were unable to establish an H2B-mTurq expressing cell line model in LNCaP, and thus they are not included in these figures. Statistics were calculated using a pairwise Mann–Whitney U test where *p < 0.1; **p < 0.01; ***p < 0.001. Pairwise statistical comparisons across all groups for average speed, MSD slopes, and inverse tortuosity may be found in Supplementary Tables S6, S7, and S8, respectively.

Tracking the motility of prostate cancer cell lines over an extended time period revealed unique stiffness-dependent characteristics. The most pronounced changes across the stiffness range occur in PC3 monolayers, which demonstrated an increase in trajectory ranges from 1–12 kPa (Figure 3A). Plotting the cell mean squared displacement (MSD) as a function of time lag, we observed an upward shift of the PC3 curves relative to less metastatic cells, indicating faster cell movement (Figure 3B). This was confirmed upon calculating the individual cell speeds, revealing that PC3 increased their median speed from 1 to 12 kPa before plateauing from 12 to 50 kPa (Figure 3E). The slope of the MSD plots reflects the nature of cell motion, where a slope of >1, 1, and <1 indicates directed (persistent), random, and confined motion, respectively (Panzetta et al., 2020). We identify a growing proportion of MSDs with slopes >1 as the microenvironment is stiffened specifically for PC3 (Figures 3C,D,F). This indicates a growing number of cells with more ballistic, directed migration behavior associated with higher metastatic potential and directed cell migration (Huda et al., 2018; Liu et al., 2020a). Moreover, the directional persistence of PC3 increases on higher substrates, as indicated by the increase in PC3’s inverse tortuosity, showing that these cells take relatively straight paths (Figure 3G). Altogether, among the three cell types, PC3 most consistently exhibits relatively high directedness (MSD slope), directional persistence (tortuosity), and speed across all stiffnesses (Supplementary Figure S3). It is to be noted that despite the variability in cell density observed across the different cell types (Supplementary Videos S1, S2, S3, and S4), we identified no significant trends in migration (as indicated by cell speed and directional persistence) or contractility (indicated by traction stress) as a function of cell density in our study (Supplementary Figure S4).

The stiffness-dependent behavior of PC3 is unique among the measured cell types. Interestingly, 22RV1 displayed an inverse relationship between substrate stiffening and the inverse tortuosity (Figure 3G), with median values decreasing as a function of substrate stiffness and the most linear trajectories being observed at lower stiffnesses. We also identify that on stiffer substrates, 22RV1 develops more confined motion as indicated by the slight leftward shift in the proportion of cells with MSD slopes less than unity (Figures 3C,D,F). This progressive inverse trend was not apparent in the speed data (Figure 3E). Surprisingly, DU145 appeared relatively insensitive to substrate stiffness in terms of its speed and tortuosity. In addition, despite the relatively low speed measured across all conditions for DU145, the proportion of cells undergoing persistent directed motion (MSD slope >1) was consistently higher than 22RV1 and comparable to PC3 (Figures 3E,F; Supplementary Figure S3). This result suggests that the collective migration of DU145 may be relatively slow but nonetheless persistent. Upon comparing these diverse migration behaviors to the contractility measurements made in Figure 1, the contractility magnitude does not appear to correspond with a particular speed or persistent motility phenotype. This suggests that contractility alone does not predict PCa motility for complete monolayers.

## 4 Discussion

In this study, we quantitatively characterize the biophysical differences of increasingly metastatic prostate cancer cell monolayers as a function of substrate stiffness in terms of their contractility, stiffness, and motility. Our results demonstrate that monolayers composed of PCa cell lines of increasing metastatic potential have distinct and unique mechanical profiles in response to variable substrate stiffnesses, where their magnitudes and sensitivities do not appear to scale with the metastatic potential in a proportional or monotonic manner. Rather, these results indicate that prostate cancer cell biophysics are highly dependent on their surrounding microenvironment, which we suggest may confer different migratory advantages and disadvantages throughout the metastatic progression. In support of this notion, PC3 and LNCaP have been shown to undergo entirely different patterns of metastasis that are uniquely induced by stiff and soft microenvironments, respectively (Liu et al., 2020b). Such findings bolster our suggestion that the mechanical uniqueness of the cell lines investigated here are reflective of the mechanical adaptations of the cell line source as opposed to universal adaptations which may arise as PCa progresses and increases its metastatic potential.

Specifically, we find that relatively aggressive cells (DU145 and PC3) exert higher contractile stresses and do more work in physiological and tumor-like stiffnesses than the tumorigenic and lowly metastatic lines (22RV1 and LNCaP, respectively). Nevertheless, contractility as measured by TFM does not absolutely predict the metastatic potential. Changes in contractile work in stiffer microenvironments may affect their cell migration and overall phenotype. We therefore speculate that cell lines may inherit mechanosensitivity based on the mechanics of their source tissue (Liu et al., 2020b). As such, there may exist stiffness thresholds whereafter cells cannot or otherwise do not exert the contractile work necessary to deform their surrounding environment. Analogously, multicellular assemblies of cardiomyocytes were found to have an optimal coupling between cell metabolism and contractile work when plated on a narrow range of intermediate physiological stiffnesses (Pasqualini et al., 2018). Although this is a vastly different cell model, this concept may have relevance in other contractile cells including PCa. Indeed, the energetic costs associated with cell-induced substrate displacement have been identified as a major factor determining the migratory path of confined breast cancer cells, which in turn is regulated by both cell and substrate stiffnesses (Zanotelli et al., 2019). A similar regulation may apply to PCa, where resident cells must mechanically interact not only with the substrate but also with their neighboring cells. Indeed, cooperative cell forces have been shown to inform collective cell migration for diverse cell types, whereby cells migrate along directions of minimal intercellular shear stress (Tambe et al., 2011).

With respect to our cell stiffness observations, we find that PCa displays cell stiffness trends both typical—that is, decreasing with metastatic potential—and atypical of conventional cancer biophysics. Interestingly, the atypical trend of PCa increasing cell stiffness with metastatic potential is consistent with previous reports (Bastatas et al., 2012; Liu et al., 2019). This suggests that additional mechanisms in PCa may regulate its biophysical changes. We identify one factor determining this trend to be the substrate stiffness, again implicating the importance of the mechanical microenvironment in dictating the biophysics of PCa cells. Furthermore, the stiffness results observed for LNCaP illustrate the potential interaction between cell stiffness and contractility. We found that LNCaP displays both higher contractility and cell stiffness from 3 to 12 kPa, revealing a unique stiffness range over which LNCaP can tune its biophysical properties. We anticipate that this dynamic change in LNCaP apparent moduli may be due to the role of contractility-mediated cytoskeletal prestress, which is known to increase dynamic cell stiffness *in situ* (Chowdhury et al., 2021)*.* For higher substrate stiffnesses, we speculate that LNCaP may reduce its cytoskeletal prestress, resulting in a decrease in cell stiffness. Previous studies have suggested that LNCaP has a reduced ability to integrate mechanical signals on stiff substrates (Liu et al., 2020b). This mechanosensitivity may be in part why LNCaP showed improved metastatic efficiency in a mouse model and expression of EMT markers on soft substrates rather than stiff substrates as PC3 does (Liu et al., 2020b). However, the said study only observed LNCaP and PC3 cells precultured on 0.7–50 kPa, whereas we reveal differences in the contractile and cortical biophysics at intermediate stiffness ranges that are more reflective of the transition from a healthy tissue to a stiffened tumor microenvironment (3–12 kPa) (Hoyt et al., 2008). A separate study comparing parental LNCaP to its more metastatic derivative cell lines (CL-1, CL-2) suggested that the apparent increase in cell stiffness as a function of metastatic potential may be due to better adhesion and focal adhesion-assisted cytoskeletal tension (Bastatas et al., 2012), which our LNCaP monolayer may have achieved by being plated on intermediate stiffness.

We observed larger heterogeneity in cell morphologies of PC3 than in more cohesive 22RV1, LNCaP, and DU145. Increased biophysical heterogeneity in response to matrix stiffening may be a critical property that enables differential migratory abilities during PCa progression. On stiffer substrates, this morphology also coincided with more directed motion and low tortuosity within a monolayer. We speculate that directed motility may present a favorable phenotype for tissue invasion (Huda et al., 2018; Liu et al., 2020a). Metastatic cells, including PC3, have been shown to exhibit significant heterogeneity in their adhesion strength, where strongly adherent metastatic cells exhibit less migratory behavior (Fuhrmann et al., 2017). Furthermore, weakly adherent PC3 cells paradoxically exert higher traction forces, and the two adhesion strength populations had opposite durotactic behaviors when confronted with a stiffness gradient (Yeoman et al., 2021). Although we do not attribute the motility differences observed in our current study to a specific mechanism, we do conclude that sensitivity to substrate stiffness is not necessarily dependent on metastatic potential alone, as DU145’s speed and directedness do not appear to outpace those of the noninvasive 22RV1 across all stiffnesses.

By considering complete PCa monolayers, this study distinguishes itself from other 2D TFM-based characterizations of cancer cell migration and contractility, which have predominantly been completed on single cells as opposed to multicellular collectives (Lintz et al., 2017; Lekka et al., 2021). The added complexity of mechanical cell–cell interactions and mechanical heterogeneity within the monolayer provide a challenge in attributing contractile observations to specific cells and their stiffnesses or migration trends. However, our multicellular characterization provides a valuable insight into a more physiologically relevant system. Cell–cell interaction is known to influence PC3 cell migration in a 3D matrix (Cui and Yamada, 2013), and both cell–cell contact and cell clustering were found to be critical for LNCaP metastasis and the associated upregulation of EMT markers (Liu et al., 2020b). This suggests that mechanical interactions between neighboring cells may be extremely consequential during PCa progression.

In addition to the mechanics considered here, the environment architecture (i.e., 3D vs 2D) and the adhesive ligand are also key factors which have been shown to modulate PCa biophysics, which may lead to deviations in trends from those reported here. With respect to the architecture, matrix stiffening differentially affects PC3’s cytoplasmic stiffness in 2D or 3D collagen networks (Baker et al., 2009), and LNCaP shows stark changes in the morphology and the organization of its cytoskeleton between 2D or 3D culture (Sieh et al., 2012). Indeed, cell geometry and orientation are established determinants of cell mechanics (Oakes et al., 2014)_._ In the comparison of metastatic and non-tumorigenic breast cancer cell motility, migration speed and persistence trends depended on the dimensionality of the motility assay (Agus et al., 2013). Therefore, connecting 2D biophysical characterizations to 3D *in vitro* or *in vivo* migration trends should be done with caution and with the consideration of the several environmental factors that may affect PCa biophysics, mechanosensing, confinement, and the motility mode. With respect to the role of adhesive ligand, throughout PCa progression, PCa cells lines may express different integrins—the receptors which selectively recognize and bind to the extracellular matrix—which therefore may attenuate or potentiate the cell–substrate mechanical coupling and biophysical response thereof (Goel et al., 2008; Sutherland et al., 2012). For example, increased expression of collagen I-binding integrin α2β1 is correlated with *in vivo* bone metastases (Sottnik et al., 2013). Interestingly, one study developed a collagen I-binding LNCaP (the parental line being non-binding) through selective culture on collagen I, resulting in an increased α2β1 expression, increased invasiveness, and the acquired ability to metastasize to bone (Hall et al., 2006, 2008). This enhanced invasive phenotype was enabled by a collagen/α2β1-mediated activation of upstream actomyosin contractility regulator RhoC (Hall et al., 2006, 2008). Likewise, PC3 has been found to show increased adhesion and cell stiffness when plated on collagen I, whereas parental LNCaP does not (Docheva et al., 2010), in part due to its intrinsically low adhesion (Liberio et al., 2014) and lack of the requisite integrin receptors (Hall et al., 2006). This ligand-dependent spreading and cell stiffening is consistent with contractility leading to PCa’s stiffening trends as a function of metastatic potential on stiff substrates with high traction stresses, but softening on soft substrates with lower stresses. Evidently, the metastasizing cells’ diverse extracellular environment and their own substrate-sensing proteome may be a major determinant of the biophysical phenotypes that arise throughout prostate cancer progression, which may be more diverse *in vivo* than those reported here for monolayers plated on fibronectin. As cells’ biophysics are influenced by both biochemical and mechanical changes of metastasis, as well as the microenvironment, it remains to be determined how these two cell regulatory aspects interact to set the cellular properties. Nevertheless, other substrate-independent factors may contribute to this behavior, as stiffening with increased metastatic potential has also been observed for PCa cells in suspension (Liu et al., 2019).

Our findings carry practical implications for the characterization of cells *in vitro*, as many measurement techniques are conducted on cells plated on glass substrates with supraphysiological stiffnesses (Faria et al., 2008; Bastatas et al., 2012; Lekka et al., 2021), or in mechanophenotyping platforms, such as microfluidic devices that use suspended cells and employ non-physiological loading rates (Liu et al., 2019). Although these established techniques can identify cancerous cell populations, we emphasize that contractility and cell stiffness are not absolute predictors of PCa metastatic potential, as these biophysical responses of these cells do not scale with one another. Moreover, this work demonstrates that different mechanical microenvironments signal diverse changes in their biophysics, and must be considered when incorporating biophysical markers to create a comprehensive physical cell “fingerprint.” We suggest that future characterizations may ideally work within an isogenic cell line with traceable modifications in their biology and metastatic potential. Although bone is the most common metastatic site for prostate cancer (Gandaglia et al., 2014), and PC3 is the most mechanically characterized PCa cell line in the literature, we suggest that PC3 is not necessarily a representative of the biophysics of all forms of metastatic PCa. Instead, we suggest that the independent mechanical changes observed are symptomatic of not only their metastatic potential and the substrate stiffness on which they were examined but additionally the biophysics that explicitly enabled their establishment at their metastatic site.

## Conclusion

In conclusion, we clarify that the cells lines commonly used to model prostate cancer metastasis and progression are mechanically distinct. Most importantly, PCa migratory behaviors, contractility, and modulation of cell stiffnesses are differentially influenced by substrate stiffness. We speculate that progressing PCa may have unique dynamic ranges of stiffness and/or contractility, over which they differentially tune their biophysical properties. These findings illustrate the importance of thoroughly characterizing the mechanics of cancer cells in their physiologically relevant mechanical microenvironments such that a more reliable catalog of contractile force-dependent biomarkers can be properly identified. Future work seeking to characterize the biophysics of prostate cancer should take into account the PCa mechanical milieu in which the cells exist *in vivo* during metastatic progression, and that observations of cells within their physiological mechanical niche may yield more accurate interpretations of the biophysical mechanisms facilitating PCa progression.

## Data Availability

The raw data supporting the conclusion of this article will be made available by the authors upon request, without undue reservation.
