# Supplementary material for: Prostate cancer cells of increasing metastatic potential exhibit diverse contractile forces, cell stiffness, and motility in a microenvironment stiffness-dependent manner
Source: Front Cell Dev Biol. 2022 Sep 19;10:932510. doi: 10.3389/fcell.2022.932510 (PMC9527313; doi:10.3389/fcell.2022.932510)
Supplement: Supplementary file 4 [file DataSheet1.docx]

Supplementary Material

# Supplementary Movies, Figures, and Tables

## Supplementary Movies

**Supplementary Movie 1.** Motility of 22RV1 monolayers over a time course of several hours imaged using confocal microscopy. Cells (green) were fluorescently labelled using CellTracker Orange, and nuclei (red) were visualized by stable expression of mTurquoise-H2B histone protein. Cell-induced fiduciary bead displacements were measured simultaneously to determine monolayer contractility. Time stamps in the top left are in units (hours:minutes:seconds). Scale bar is 50 microns.

**Supplementary Movie 2.** Motility of LNCaP monolayers over a time course of several hours imaged using confocal microscopy. Cells (green) were dyed with CellTracker Orange. LNCaP with mTurquoise-labelled nuclei were not generated for this manuscript. Cell-induced fiduciary bead displacements were measured simultaneously to determine monolayer contractility. Time stamps in the top left are in units (hours:minutes:seconds). Scale bar is 50 microns.

**Supplementary Movie 3.** Motility of DU145 monolayers over a time course of several hours imaged using confocal microscopy. Cells (green) were fluorescently labelled using CellTracker Orange, and nuclei (red) were visualized by stable expression of mTurquoise-H2B histone protein. Cell-induced fiduciary bead displacements were measured simultaneously to determine monolayer contractility. Time stamps in the top left are in units (hours:minutes:seconds). Scale bar is 50 microns.

**Supplementary Movie 4.** Motility of PC3 monolayers over monolayers over a time course of several hours imaged using confocal microscopy. Cells (green) were fluorescently labelled using CellTracker Orange, and nuclei (red) were visualized by stable expression of mTurquoise-H2B histone protein. Cell-induced fiduciary bead displacements were measured simultaneously to determine monolayer contractility. Time stamps in the top left are in units (hours:minutes:seconds). Scale bar is 50 microns.

## Supplementary Figures

**
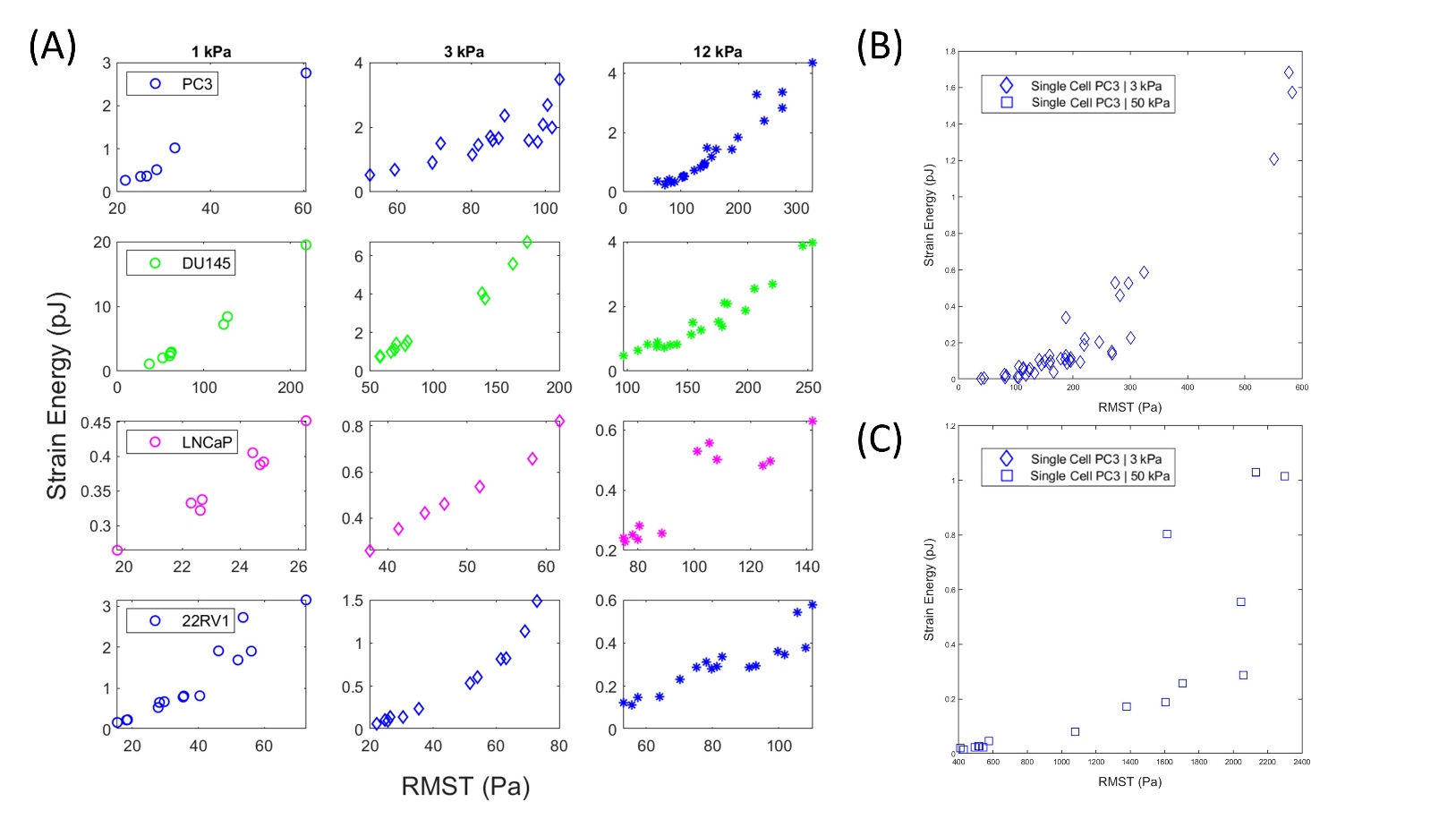
**

**Supplementary Figure 1.** Strain Energy is positively correlated with the RMST for all monolayers in a given condition. This is distinct from the non-correlated relationship between RMST and Strain energy across substrates of increasing stiffness. **(A)** Strain Energy as a function of RMST data points presented in Figures E-H, demonstrating a conserved positive correlation when comparing tractions and strain energy within a given sample. **(B,C)** Single PC3 cells on 3kPa **(B)** and **(C)** 50 kPa substrates are positively correlated, as in the monolayer case. Single cell contractility measurements consisted of a single time point.


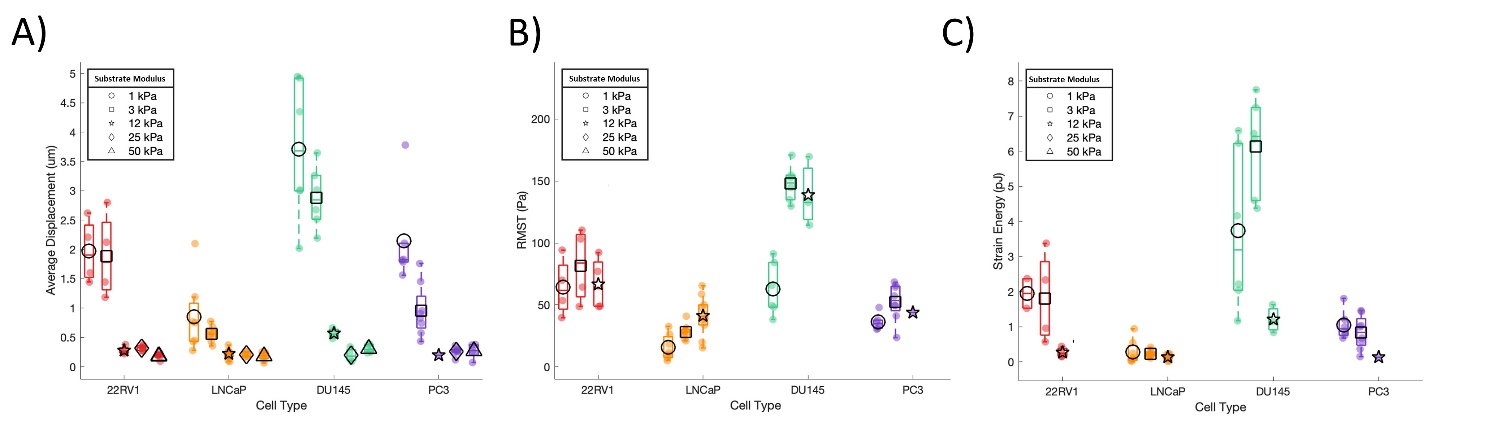


**Supplementary Figure 2.** Contractility of prostate cancer monolayers measured for cortical stiffness measurements using OMTC. **(A)** Average monolayer-induced surface deformations, **(B)** Root Mean Squared (average) Traction Stress (RMST) and **(C)** strain energy of PCa monolayers cultured on substrates of various stiffness. Contractile outputs of RMST and strain energy on 25 kPa and 50 kPa are omitted due to their displacements converging to the noise floor, yielding with traction below the resolution of these measurements. OMTC experiments were conducted at 37 deg C with atmospheric CO_2_ content within 1 hour of removal from the incubator (held at 5% CO_2_).


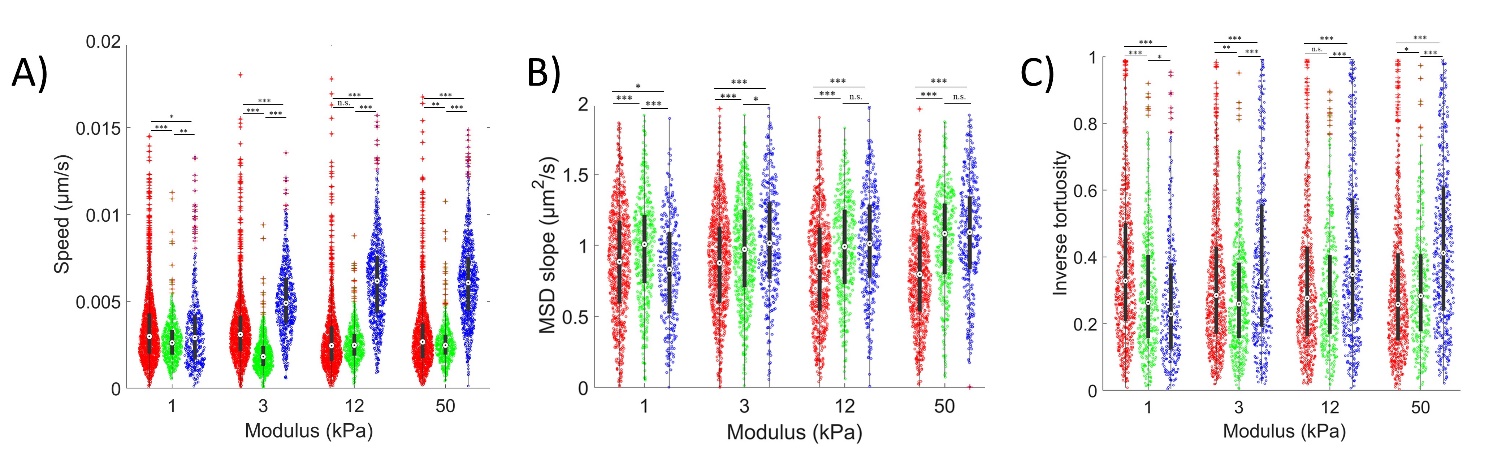


**Supplementary Figure 3. (A)** Average PCa cell speed, **(B)** MSD slope (persistence of motion), and **(C)** Inverse tortuosity as shown in Figure 3E-G here grouped according to substrate stiffness to compare differences between cell types. MSD slopes indicate the nature of cell motion, where slope > 1 indicates directed motion, 1 indicates random motion, and <1 indicates confined motion. Inverse tortuosity indicates the average persistence of motion along of a given cell trajectory, where 1 indicates a completely straight path. We were unable to establish an H2B-mTurq expressing cell line model in LNCaP, and thus they are not included in these figures. Planned statistical comparisons were performed using a pairwise Mann-Whitney U test where **p<*0.1; ** *p<*0.01; *** *p<*0.001.


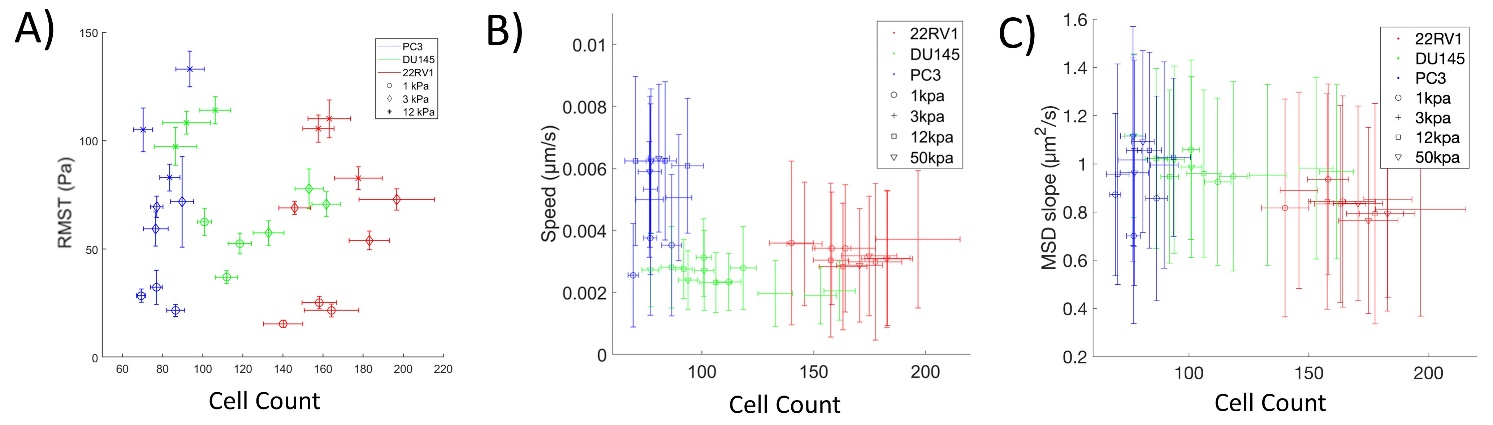


**Supplementary Figure 4.** Prostate cancer cell contractility and motility, respectively indicated by traction stress and average cell speed, does not vary significantly with cell density. **(A)** Time-averaged Root-mean-square traction stress as a function of average cell number per field. The RMST values reported here correspond to the motility results reported in Figure 3 and Supplementary Figure 3. **(B)** Average cell speed as a function cell count per field. For all experimental groups, although different cell types have a tendency to cluster at different densities, RMST and cell speed do not show a significant trend within a given stiffness condition for 22RV1, DU145, and PC3. We were unable to establish an H2B-mTurq expressing cell line model in LNCaP, and thus the density dependent effects or lack thereof were not calculated. Error bars in the cell count indicate the standard deviation in cell count per-field for a given time course. Error bars in panel (A) indicate the standard deviation of RMST fluctuations over the acquisition time course. Error bars in panels (B) and (C) indicate the standard deviation of the distributions shown in Figures 3E and 3F, respectively.

## Supplementary Tables

**Supplementary Table 1**: Previous reports characterizing the metastatic potential of Prostate Cancer Cell lines used in this study. Classical definitions are primarily based on *in vivo* observations of metastatic ability, but these are often reflected in *in vitro* migration and invasion assays. Though relative invasiveness between PC3 and DU145 are varied *in vitro*.

| Assay/model | Cell lines compared | Observation | Reference |
| --- | --- | --- | --- |
| *in vitro* |  |  |  |
| Migration  and/or  Invasion Assay  (Boyden Chamber, Matrigel,  Porous boundary,  etc.) | PC3 and DU145 | PC3 Migration and Invasiveness > DU145 | A. et al., 1987  Fang et al., 2017  Colella et al., 2004 |
|  |  |  |  |
|  | DU145 and. LNCaP | Migration of DU145 > LNCaP | Chunthapong et al., 2004 |
|  |  |  |  |
|  | PC3 and LNCaP | Invasion rate of PC3 > LNCaP | Laniado et al., 1997 |
|  |  |  |  |
|  | PC3, DU145, and LNCaP | Invasion rate of PC3 > DU145 > LNCaP | Aalinkeel et al., 2004 |
|  |  | Invasion rate of DU145 > PC3 >> LNCaP | Moroz et al., 2013 |
|  |  |  |  |
| Matrigel Invasion and Wound Closure Assays | LNCaP and 22RV1 | LNCaP Invasiveness approximately equal to 22RV1; 22RV1 migration rate > LNCaP | Dai et al., 2019 |
|  |  |  |  |
| 3D Matrigel Drop Invasion | PC3, DU145, LNCaP, & 22RV1 | Invasion area of DU145 > PC3 > 22RV1 > LNCaP | Aslan et al., 2021 |
|  |  |  |  |
| *in vivo* |  |  |  |
| Cells Injected or implanted into Nude Mice |  |  |  |
|  | PC3 and DU145 | Incidence and spread of metastasis to other organs of PC3 > DU145 | Kozlowski et al., 1984  Bastide et al., 2002 |
|  |  |  |  |
|  | PC3 and LNCaP | Tumorigenicity and incidence of metastasis of PC3 > LNCaP | Rembrink et al., 1997  Stephenson et al., 1992 |
|  |  |  |  |
|  |  | Tumorigenicity and metastatic efficiency PC3 > LNCaP when pre-cultured on stiff substrates; reversed with soft pre-culture | Liu et al., 2020 |
|  |  |  |  |
|  | 22RV1 and PC3 | 22RV1 more tumorigenic than PC3-LNC4 (PC3-derived) but not metastatic | Kovar et al., 2006 |

**Supplementary Table 2.** Statistical results (p-values) obtained from pairwise comparisons of time-averaged TFM bead displacements across all cell lines and stiffnesses. Select comparisons across stiffnesses for a single cell type are shown in Figure 1D. All p-values were calculated using a Mann-Whitney U test for the planned comparisons indicated in the corresponding figure. Red, yellow, and green shading respectively indicate the significance thresholds: **p<*0.1; ** *p<*0.01; *** *p<*0.001.

| Cell Type | - | 22RV1 | | | LNCaP | | | DU145 | | | PC3 | | |
| --- | --- | --- | --- | --- | --- | --- | --- | --- | --- | --- | --- | --- | --- |
| \| | Stiffness | 1 kPa | 3 kPa | 12 kPa | 1 kPa | 3 kPa | 12 kPa | 1 kPa | 3 kPa | 12 kPa | 1 kPa | 3 kPa | 12 kPa |
| 22RV1 | 1 kPa | 1 |  |  |  |  |  |  |  |  |  |  |  |
|  | 3 kPa | 3E-04 | 1 |  |  |  |  |  |  |  |  |  |  |
|  | 12 kPa | 3E-06 | 7E-06 | 1 |  |  |  |  |  |  |  |  |  |
| LNCaP | 1 kPa | 0.022 | 0.019 | 8E-05 | 1 |  |  |  |  |  |  |  |  |
|  | 3 kPa | 4E-04 | 0.227 | 2E-04 | 6E-04 | 1 |  |  |  |  |  |  |  |
|  | 12 kPa | 2E-05 | 4E-05 | 0.642 | 2E-04 | 4E-05 | 1 |  |  |  |  |  |  |
| DU145 | 1 kPa | 0.003 | 2E-04 | 8E-05 | 2E-04 | 3E-04 | 2E-04 | 1 |  |  |  |  |  |
|  | 3 kPa | 0.19 | 6E-04 | 7E-06 | 0.019 | 4E-05 | 4E-05 | 2E-04 | 1 |  |  |  |  |
|  | 12 kPa | 0.178 | 0.01 | 2E-07 | 0.431 | 0.004 | 3E-06 | 6E-04 | 0.654 | 1 |  |  |  |
| PC3 | 1 kPa | 0.343 | 0.01 | 4E-04 | 0.755 | 0.001 | 1E-04 | 0.005 | 0.666 | 0.738 | 1 |  |  |
|  | 3 kPa | 0.048 | 9E-04 | 1E-06 | 0.062 | 6E-04 | 9E-06 | 1E-04 | 0.531 | 0.787 | 0.971 | 1 |  |
|  | 12 kPa | 9E-07 | 0.002 | 6E-06 | 1E-04 | 0.069 | 1E-04 | 3E-05 | 2E-06 | 2E-06 | 5E-04 | 6E-07 | 1 |

**Supplementary Table 3.** Statistical results (p-values) obtained from pairwise comparisons of time-averaged root mean-squared traction (RMST) stress across all cell lines and stiffnesses. Select comparisons are shown in Figures 1E,F. All p-values were calculated using a Mann-Whitney U test for the planned comparisons indicated in the corresponding figures. Red, yellow, and green shading respectively indicate the significance thresholds: **p<*0.1; ** *p<*0.01; *** *p<*0.001.

| Cell Type | - | 22RV1 | | | LNCaP | | | DU145 | | | PC3 | | |
| --- | --- | --- | --- | --- | --- | --- | --- | --- | --- | --- | --- | --- | --- |
| \| | Stiffness | 1 kPa | 3 kPa | 12 kPa | 1 kPa | 3 kPa | 12 kPa | 1 kPa | 3 kPa | 12 kPa | 1 kPa | 3 kPa | 12 kPa |
| 22RV1 | 1 kPa | 1 |  |  |  |  |  |  |  |  |  |  |  |
|  | 3 kPa | 0.425 | 1 |  |  |  |  |  |  |  |  |  |  |
|  | 12 kPa | 1E-05 | 2E-04 | 1 |  |  |  |  |  |  |  |  |  |
| LNCaP | 1 kPa | 0.032 | 0.004 | 8E-05 | 1 |  |  |  |  |  |  |  |  |
|  | 3 kPa | 0.093 | 0.711 | 0.0008 | 3E-04 | 1 |  |  |  |  |  |  |  |
|  | 12 kPa | 2E-05 | 4E-05 | 0.138 | 2E-04 | 4E-05 | 1 |  |  |  |  |  |  |
| DU145 | 1 kPa | 0.003 | 0.041 | 0.6622 | 2E-04 | 0.054 | 0.2318 | 1 |  |  |  |  |  |
|  | 3 kPa | 5E-05 | 6E-04 | 0.9118 | 2E-04 | 5E-04 | 0.2854 | 0.297 | 1 |  |  |  |  |
|  | 12 kPa | 1E-06 | 3E-06 | 6E-07 | 5E-05 | 1E-04 | 0.0001 | 0.006 | 0.001 | 1 |  |  |  |
| PC3 | 1 kPa | 0.483 | 0.213 | 0.0009 | 0.043 | 0.035 | 0.0001 | 0.003 | 4E-04 | 0.0003 | 1 |  |  |
|  | 3 kPa | 1E-05 | 9E-05 | 0.7322 | 1E-04 | 5E-04 | 0.2017 | 0.561 | 0.659 | 9E-07 | 8E-04 | 1 |  |
|  | 12 kPa | 5E-07 | 3E-06 | 0.0005 | 3E-05 | 1E-04 | 0.036 | 0.025 | 0.016 | 0.1753 | 3E-04 | 7E-04 | 1 |

**Supplementary Table 4.** Statistical results (p-values) obtained from pairwise comparisons of time-averaged strain energy across all cell lines and stiffnesses. Select comparisons are shown in Figures 1G,H. All p-values were calculated using a Mann-Whitney U test for the planned comparisons indicated in the corresponding figures. Red, yellow, and green shading respectively indicate the significance thresholds: **p<*0.1; ** *p<*0.01; *** *p<*0.001.

| Cell Type | - | 22RV1 | | | LNCaP | | | DU145 | | | PC3 | | |
| --- | --- | --- | --- | --- | --- | --- | --- | --- | --- | --- | --- | --- | --- |
| \| | Stiffness | 1 kPa | 3 kPa | 12 kPa | 1 kPa | 3 kPa | 12 kPa | 1 kPa | 3 kPa | 12 kPa | 1 kPa | 3 kPa | 12 kPa |
| 22RV1 | 1 kPa | 1 |  |  |  |  |  |  |  |  |  |  |  |
|  | 3 kPa | 0.054 | 1 |  |  |  |  |  |  |  |  |  |  |
|  | 12 kPa | 0.002 | 0.773 | 1 |  |  |  |  |  |  |  |  |  |
| LNCaP | 1 kPa | 0.032 | 1 | 0.0756 | 1 |  |  |  |  |  |  |  |  |
|  | 3 kPa | 0.168 | 0.65 | 0.0188 | 0.094 | 1 |  |  |  |  |  |  |  |
|  | 12 kPa | 0.015 | 1 | 0.364 | 0.969 | 0.227 | 1 |  |  |  |  |  |  |
| DU145 | 1 kPa | 0.002 | 4E-04 | 8E-05 | 2E-04 | 3E-04 | 0.0002 | 1 |  |  |  |  |  |
|  | 3 kPa | 0.054 | 0.001 | 7E-06 | 2E-04 | 2E-04 | 4E-05 | 0.082 | 1 |  |  |  |  |
|  | 12 kPa | 0.128 | 8E-04 | 3E-07 | 5E-05 | 7E-04 | 8E-06 | 0.007 | 0.34 | 1 |  |  |  |
| PC3 | 1 kPa | 0.536 | 0.385 | 0.0389 | 0.282 | 0.836 | 0.2129 | 0.008 | 0.017 | 0.0479 | 1 |  |  |
|  | 3 kPa | 0.092 | 2E-04 | 2E-06 | 1E-04 | 6E-04 | 2E-05 | 0.009 | 0.908 | 0.3813 | 0.036 | 1 |  |
|  | 12 kPa | 0.797 | 0.016 | 1E-05 | 0.003 | 0.069 | 0.0019 | 0.003 | 0.036 | 0.1226 | 0.265 | 0.04 | 1 |

**Supplementary Table 5:** Statistical results (p-values) obtained from pairwise comparisons of cell apparent moduli measured using OMTC across all cell lines and stiffnesses. Select comparisons are shown in Figures 2D,E. All p-values were calculated using a Mann-Whitney U test for the planned comparisons indicated in the corresponding figures. Red, yellow, and green shading respectively indicate the significance thresholds: **p<*0.1; ** *p<*0.01; *** *p<*0.001.

| Cell Type | - | 22RV1 | | | | | LNCaP | | | | |
| --- | --- | --- | --- | --- | --- | --- | --- | --- | --- | --- | --- |
| \| | Stiffness | 1 kPa | 3 kPa | 12 kPa | 25 kPa | 50 kPa | 1 kPa | 3 kPa | 12 kPa | 25 kPa | 50 kPa |
| 22RV1 | 1 kPa | 1 |  |  |  |  |  |  |  |  |  |
|  | 3 kPa | 0.001 | 1 |  |  |  |  |  |  |  |  |
|  | 12 kPa | 0.002 | 0.484 | 1 |  |  |  |  |  |  |  |
|  | 25 kPa | 0.017 | 0.895 | 0.6174 | 1 |  |  |  |  |  |  |
|  | 50 kPa | 6E-05 | 0.004 | 0.006 | 0.0359 | 1 |  |  |  |  |  |
| LNCaP | 1 kPa | 0.775 | 2E-04 | 0.0007 | 0.0225 | 9E-06 | 1 |  |  |  |  |
|  | 3 kPa | 0.218 | 0.281 | 0.205 | 0.426 | 0.0038 | 0.398 | 1 |  |  |  |
|  | 12 kPa | 2E-06 | 2E-06 | 5E-06 | 0.0009 | 0.1475 | 3E-08 | 2E-04 | 1 |  |  |
|  | 25 kPa | 0.439 | 0.105 | 0.2077 | 0.23 | 0.0094 | 0.838 | 0.682 | 0.0008 | 1 |  |
|  | 50 kPa | 0.94 | 2E-04 | 0.0003 | 0.014 | 6E-07 | 0.532 | 0.228 | 3E-09 | 0.6738 | 1 |
| DU145 | 1 kPa | 0.991 | 0.004 | 0.0046 | 0.0422 | 0.0004 | 0.636 | 0.457 | 6E-05 | 0.7814 | 0.9651 |
|  | 3 kPa | 0.914 | 0.013 | 0.0145 | 0.0685 | 0.0003 | 0.865 | 0.364 | 2E-05 | 0.8053 | 0.7301 |
|  | 12 kPa | 0.565 | 1E-03 | 0.0031 | 0.0092 | 8E-06 | 0.176 | 0.046 | 4E-08 | 0.1632 | 0.1931 |
|  | 25 kPa | 0.321 | 0.167 | 0.2244 | 0.3418 | 0.0354 | 0.447 | 0.955 | 0.0155 | 0.782 | 0.3309 |
|  | 50 kPa | 0.078 | 0.048 | 0.0651 | 0.2712 | 8E-05 | 0.078 | 0.894 | 3E-08 | 0.624 | 0.023 |
| PC3 | 1 kPa | 0.181 | 1E-04 | 0.0013 | 0.0024 | 3E-05 | 0.058 | 0.013 | 8E-07 | 0.0311 | 0.0564 |
|  | 3 kPa | 0.893 | 0.035 | 0.0706 | 0.1092 | 0.0057 | 0.835 | 0.364 | 0.0011 | 0.6825 | 0.8605 |
|  | 12 kPa | 0.794 | 0.003 | 0.0097 | 0.0507 | 9E-05 | 0.743 | 0.455 | 2E-06 | 0.9875 | 0.8338 |
|  | 25 kPa | 0.03 | 0.561 | 0.4127 | 0.7611 | 0.0075 | 0.031 | 0.473 | 8E-05 | 0.3319 | 0.0156 |
|  | 50 kPa | 0.004 | 0.994 | 0.6778 | 0.9438 | 0.0058 | 0.001 | 0.153 | 1E-05 | 0.0972 | 0.0002 |

| Cell Type | - | DU145 | | | | | PC3 | | | | |
| --- | --- | --- | --- | --- | --- | --- | --- | --- | --- | --- | --- |
| \| | Stiffness | 1 kPa | 3 kPa | 12 kPa | 25 kPa | 50 kPa | 1 kPa | 3 kPa | 12 kPa | 25 kPa | 50 kPa |
| DU145 | 1 kPa | 1 |  |  |  |  |  |  |  |  |  |
|  | 3 kPa | 0.906 | 1 |  |  |  |  |  |  |  |  |
|  | 12 kPa | 0.351 | 0.25 | 1 |  |  |  |  |  |  |  |
|  | 25 kPa | 0.463 | 0.407 | 0.1313 | 1 |  |  |  |  |  |  |
|  | 50 kPa | 0.07 | 0.157 | 0.0253 | 0.8662 | 1 |  |  |  |  |  |
| PC3 | 1 kPa | 0.101 | 0.086 | 0.1885 | 0.0201 | 0.0057 | 1 |  |  |  |  |
|  | 3 kPa | 0.9 | 0.81 | 0.4333 | 0.4998 | 0.2624 | 0.106 | 1 |  |  |  |
|  | 12 kPa | 0.905 | 1 | 0.0861 | 0.475 | 0.1198 | 0.031 | 0.693 | 1 |  |  |
|  | 25 kPa | 0.053 | 0.082 | 0.0121 | 0.5944 | 0.347 | 0.003 | 0.188 | 0.0753 | 1 |  |
|  | 50 kPa | 0.006 | 0.01 | 0.0003 | 0.2125 | 0.1166 | 2E-04 | 0.032 | 0.0029 | 0.5862 | 1 |

**Supplementary Table 6.** Statistical results (p-values) obtained from pairwise comparisons of average cell speeds across all cell lines and stiffnesses. Select comparisons are shown in Figure 3E and Supplementary Figure 2A. All p-values were calculated using a Mann-Whitney U test for the planned comparisons indicated in the corresponding figure. Red, yellow, and green shading respectively indicate the significance thresholds: **p<*0.1; ** *p<*0.01; *** *p<*0.001.

| Cell Type | - | 22RV1 | | | | DU145 | | | | | | PC3 | | | | | |  |
| --- | --- | --- | --- | --- | --- | --- | --- | --- | --- | --- | --- | --- | --- | --- | --- | --- | --- | --- |
| \| | Stiffness | 1 kPa | 3 kPa | 12 kPa | 50 kPa | 1 kPa | 3 kPa | 12 kPa | | 50 kPa | | 1 kPa | | 3 kPa | | 12 kPa | |  |
| 22RV1 | 1 kPa | 1 |  |  |  |  |  | |  | |  | |  | |  | |  | |
|  | 3 kPa | 0.2812 | 1 |  |  |  |  | |  | |  | |  | |  | |  | |
|  | 12 kPa | 4E-20 | 5E-25 | 1 |  |  |  | |  | |  | |  | |  | |  | |
|  | 50 kPa | 1E-09 | 4E-13 | 0.0013 | 1 |  |  | |  | |  | |  | |  | |  | |
| DU145 | 1 kPa | 2E-09 | 1E-13 | 0.0183 | 0.4207 | 1 |  | |  | |  | |  | |  | |  | |
|  | 3 kPa | 6E-86 | 3E-97 | 4E-29 | 1E-46 | 2E-40 | 1 | |  | |  | |  | |  | |  | |
|  | 12 kPa | 1E-14 | 4E-20 | 0.6906 | 0.0059 | 0.027 | 1E-29 | | 1 | |  | |  | |  | |  | |
|  | 50 kPa | 2E-14 | 4E-20 | 0.8245 | 0.004 | 0.0164 | 4E-28 | | 0.8774 | | 1 | |  | |  | |  | |
| PC3 | 1 kPa | 0.0629 | 0.017 | 5E-05 | 0.0327 | 0.0077 | 3E-32 | | 1E-04 | | 8E-05 | | 1 | |  | |  | |
|  | 3 kPa | 7E-70 | 2E-72 | 9E-107 | 2E-98 | 3E-96 | 7E-160 | | 2E-99 | | 2E-96 | | 2E-44 | | 1 | |  | |
|  | 12 kPa | 6E-135 | 6E-139 | 8E-168 | 1E-162 | 3E-140 | 2E-201 | | 1E-138 | | 7E-133 | | 5E-77 | | 5E-17 | | 1 | |
|  | 50 kPa | 8E-150 | 4E-154 | 1E-184 | 2E-179 | 1E-152 | 1E-217 | | 1E-149 | | 6E-143 | | 4E-82 | | 2E-17 | | 0.6556 | |

**Supplementary Table 7:** Statistical results (p-values) obtained from pairwise comparisons of slopes obtained from the Mean Squared Displacement (MSD) of individual cell trajectories across all cell lines and stiffnesses. Select comparisons are shown in Figure 3F and Supplementary Figure 3B. All p-values were calculated using a Mann-Whitney U test for the planned comparisons indicated in the corresponding figure. Red, yellow, and green shading respectively indicate the significance thresholds: **p<*0.1; ** *p<*0.01; *** *p<*0.001.

| Cell Type | - | 22RV1 | | | | DU145 | | | | PC3 |  |  |
| --- | --- | --- | --- | --- | --- | --- | --- | --- | --- | --- | --- | --- |
| \| | Stiffness | 1 kPa | 3 kPa | 12 kPa | 50 kPa | 1 kPa | 3 kPa | 12 kPa | 50 kPa | 1 kPa | 3 kPa | 12 kPa |
| 22RV1 | 1 kPa | 1 |  |  |  |  |  |  |  |  |  |  |
|  | 3 kPa | 0.477 | 1 |  |  |  |  |  |  |  |  |  |
|  | 12 kPa | 0.062 | 0.196 | 1 |  |  |  |  |  |  |  |  |
|  | 50 kPa | 3E-04 | 0.001 | 0.1058 | 1 |  |  |  |  |  |  |  |
| DU145 | 1 kPa | 5E-05 | 7E-07 | 1E-08 | 6E-14 | 1 |  |  |  |  |  |  |
|  | 3 kPa | 4E-05 | 6E-07 | 5E-09 | 8E-15 | 0.792 | 1 |  |  |  |  |  |
|  | 12 kPa | 5E-05 | 9E-07 | 1E-08 | 2E-13 | 0.82 | 0.687 | 1 |  |  |  |  |
|  | 50 kPa | 1E-10 | 1E-13 | 2E-15 | 7E-22 | 0.007 | 0.004 | 0.0177 | 1 |  |  |  |
| PC3 | 1 kPa | 0.029 | 0.077 | 0.4886 | 0.538 | 4E-08 | 3E-08 | 3E-08 | 1E-13 | 1 |  |  |
|  | 3 kPa | 6E-08 | 5E-10 | 9E-12 | 3E-17 | 0.079 | 0.036 | 0.1074 | 0.595 | 9E-11 | 1 |  |
|  | 12 kPa | 8E-08 | 5E-10 | 8E-12 | 2E-17 | 0.118 | 0.053 | 0.1555 | 0.398 | 7E-11 | 0.811 | 1 |
|  | 50 kPa | 2E-13 | 1E-16 | 3E-18 | 2E-25 | 3E-04 | 7E-05 | 0.0008 | 0.263 | 6E-16 | 0.112 | 0.0572 |

**Supplementary Table 8:** Statistical results (p-values) obtained from pairwise comparisons of inverse tortuosity values of individual cell trajectories across all cell lines and stiffnesses. Select comparisons are shown in Figure 3G and Supplementary Figure 3C. All p-values were calculated using a Mann-Whitney U test for the planned comparisons indicated in the corresponding figure. Red, yellow, and green shading respectively indicate the significance thresholds: **p<*0.1; ** *p<*0.01; *** *p<*0.001.

| Cell Type | - | 22RV1 | | | | DU145 | | | | PC3 | | |
| --- | --- | --- | --- | --- | --- | --- | --- | --- | --- | --- | --- | --- |
| \| | Stiffness | 1 kPa | 3 kPa | 12 kPa | 50 kPa | 1 kPa | 3 kPa | 12 kPa | 50 kPa | 1 kPa | 3 kPa | 12 kPa |
| 22RV1 | 1 kPa | 1 |  |  |  |  |  |  |  |  |  |  |
|  | 3 kPa | 1E-05 | 1 |  |  |  |  |  |  |  |  |  |
|  | 12 kPa | 3E-05 | 0.822 | 1 |  |  |  |  |  |  |  |  |
|  | 50 kPa | 2E-10 | 0.016 | 0.0523 | 1 |  |  |  |  |  |  |  |
| DU145 | 1 kPa | 7E-09 | 0.047 | 0.095 | 0.8839 | 1 |  |  |  |  |  |  |
|  | 3 kPa | 9E-12 | 0.005 | 0.0209 | 0.7284 | 0.573 | 1 |  |  |  |  |  |
|  | 12 kPa | 6E-06 | 0.418 | 0.5769 | 0.2033 | 0.309 | 0.106 | 1 |  |  |  |  |
|  | 50 kPa | 9E-05 | 0.83 | 0.9832 | 0.0713 | 0.121 | 0.027 | 0.5814 | 1 |  |  |  |
| PC3 | 1 kPa | 7E-13 | 3E-05 | 0.0002 | 0.0254 | 0.025 | 0.052 | 0.0017 | 0.0005 | 1 |  |  |
|  | 3 kPa | 0.83 | 3E-04 | 0.0006 | 2E-07 | 1E-06 | 2E-08 | 0.0001 | 0.001 | 8E-10 | 1 |  |
|  | 12 kPa | 0.064 | 8E-08 | 4E-07 | 4E-12 | 1E-10 | 2E-13 | 8E-08 | 2E-06 | 5E-14 | 0.193 | 1 |
|  | 50 kPa | 2E-06 | 2E-17 | 4E-15 | 6E-23 | 2E-20 | 2E-25 | 7E-16 | 5E-14 | 9E-23 | 5E-04 | 0.0288 |

**References for Supplementary Table 1**

A., A., Y., I., & H.K., K. (1987). A rapid in vitro assay for quantitating the invasive potential of tumor cells. *Cancer Research*, *47*(12).

Aalinkeel, R., Nair, M. P. N., Sufrin, G., Mahajan, S. D., Chadha, K. C., Chawda, R. P., & Schwartz, S. A. (2004). Gene expression of angiogenic factors correlates with metastatic potential of prostate cancer cells. *Cancer Research*, *64*(15). https://doi.org/10.1158/0008-5472.CAN-2506-2

Aslan, M., Hsu, E. C., Liu, S., & Stoyanova, T. (2021). Quantifying the invasion and migration ability of cancer cells with a 3D Matrigel drop invasion assay. *Biology Methods and Protocols*, *6*(1). https://doi.org/10.1093/biomethods/bpab014

Bastide, C., Bagnis, C., Mannoni, P., Hassoun, J., & Bladou, F. (2002). A Nod Scid mouse model to study human prostate cancer. *Prostate Cancer and Prostatic Diseases*, *5*(4). https://doi.org/10.1038/sj.pcan.4500606

Chunthapong, J., Seftor, E. A., Khalkhali-Ellis, Z., Seftor, R. E. B., Amir, S., Lubaroff, D. M., Heidger, P. M., & Hendrix, M. J. C. (2004). Dual roles of E-cadherin in prostate cancer invasion. *Journal of Cellular Biochemistry*, *91*(4). https://doi.org/10.1002/jcb.20032

Colella, R., Jackson, T., & Goodwyn, E. (2004). Matrigel® invasion by the prostate cancer cell lines, PC3 and DU145, and cathepsin L+B activity. *Biotechnic and Histochemistry*, *79*(3–4). https://doi.org/10.1080/10520290400010572

Dai, X., Liang, Z., Liu, L., Guo, K., Xu, S., & Wang, H. (2019). Silencing of MALAT1 inhibits migration and invasion by sponging MIR-1-3p in Prostate cancer cells. *Molecular Medicine Reports*, *20*(4). https://doi.org/10.3892/mmr.2019.10602

Fang, L. L., Sun, B. F., Huang, L. R., Yuan, H. B., Zhang, S., Chen, J., Yu, Z. J., & Luo, H. (2017). Potent inhibition of miR-34b on migration and invasion in metastatic prostate cancer cells by regulating the TGF-β pathway. *International Journal of Molecular Sciences*, *18*(12). https://doi.org/10.3390/ijms18122762

Kovar, J. L., Johnson, M. A., Volcheck, W. M., Chen, J., & Simpson, M. A. (2006). Hyaluronidase expression induces prostate tumor metastasis in an orthotopic mouse model. *American Journal of Pathology*, *169*(4). https://doi.org/10.2353/ajpath.2006.060324

Kozlowski, J. M., Fidler, I. J., Campbell, D., Xu, Z. L., Kaighn, M. E., & Hart, I. R. (1984). Metastatic Behavior off Human Tumor Cell Lines Grown in the Nude Mouse. *Cancer Research*, *44*(8).

Laniado, M. E., Lalani, E. N., Fraser, S. P., Grimes, J. A., Bhangal, G., Djamgoz, M. B. A., & Abel, P. D. (1997). Expression and functional analysis of voltage-activated Na+ channels in human prostate cancer cell lines and their contribution to invasion in vitro. *American Journal of Pathology*, *150*(4).

Liu, Z., Wang, L., Xu, H., Du, Q., Li, L., Wang, L., Zhang, E. S., Chen, G., & Wang, Y. (2020). Heterogeneous Responses to Mechanical Force of Prostate Cancer Cells Inducing Different Metastasis Patterns. *Advanced Science*. https://doi.org/10.1002/advs.201903583

Moroz, A., Delella, F. K., Almeida, R., Lacorte, L. M., Fávaro, W. J., Deffune, E., & Felisbino, S. L. (2013). Finasteride inhibits human prostate cancer cell invasion through MMP2 and MMP9 downregulation. *PLoS ONE*, *8*(12). https://doi.org/10.1371/journal.pone.0084757

Rembrink, K., Romijn, J. C., van der Kwast, T. H., Rübben, H., & Schröder, F. H. (1997). Orthotopic implantation of human prostate cancer cell lines: A clinically relevant animal model for metastatic prostate cancer. *Prostate*, *31*(3). https://doi.org/10.1002/(SICI)1097-0045(19970515)31:3<168::AID-PROS4>3.0.CO;2-H

Stephenson, R. A., Dinney, C. P. N., Gohji, K., Ordonez, N. G., Killion, J. J., & Fidler, I. J. (1992). Metastatic model for human prostate cancer using orthotopic implantation in nude mice. *Journal of the National Cancer Institute*, *84*(12). https://doi.org/10.1093/jnci/84.12.951
